# Subcutaneous fat necrosis of the newborn with fluctuant nodules mimicking infection

**DOI:** 10.1016/j.jdcr.2024.03.019

**Published:** 2024-04-09

**Authors:** Allison Holt, Sarah Servattalab, Kaitlyn Yim, A. Yasmine Kirkorian, Patrick O’Donnell, Karen Wiss

**Affiliations:** a Department of Dermatology, UMass Chan Medical School, Worcester, Massachusetts; b Department of Pathology, UMass Memorial Medical Center, Worcester, Massachusetts; c Division of Dermatology, Children’s National Hospital, Washington, District of Columbia; d Department of Pediatrics, UMass Chan Medical School, Worcester, Massachusetts

**Keywords:** fluctuant nodules, infection, panniculitis, subcutaneous fat necrosis

## Introduction

Subcutaneous fat necrosis of the newborn (SCFN) is a panniculitis that affects term or post-term infants and manifests within the first few weeks of life. It typically appears as erythematous, indurated nodules or plaques most commonly affecting the back, buttocks, and extremities.^1^^,^^2^ The lesions are classically noted to be firm and are rarely associated with drainage; however, several previous reports have described cases of fluctuant and draining lesions comparable to abscesses.^3^, ^4^, ^5^ Here, we report 2 cases of SCFN with purulent lesions initially concerning for an infectious process whose biopsies demonstrated findings of SCFN.

## Case reports

### Case 1

**Fig 1:**
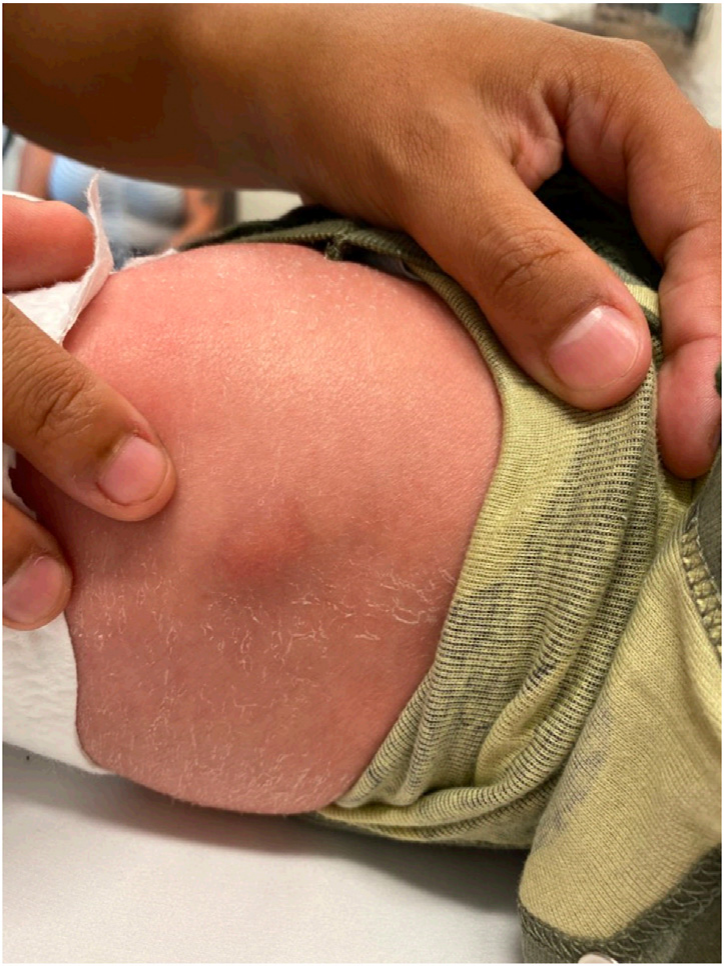
Fluctuant subcutaneous nodule located to the left of the midspine on a 14-day-old infant.

**Fig 2:**
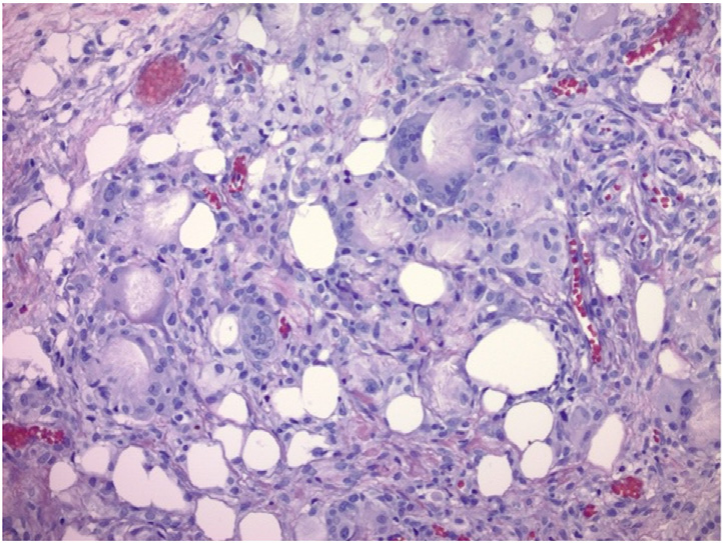
Necrotic adipocytes with radiating needle-shaped crystalline material within, admixed with granulomatous inflammation (H&E, ×200).

**Fig 3:**
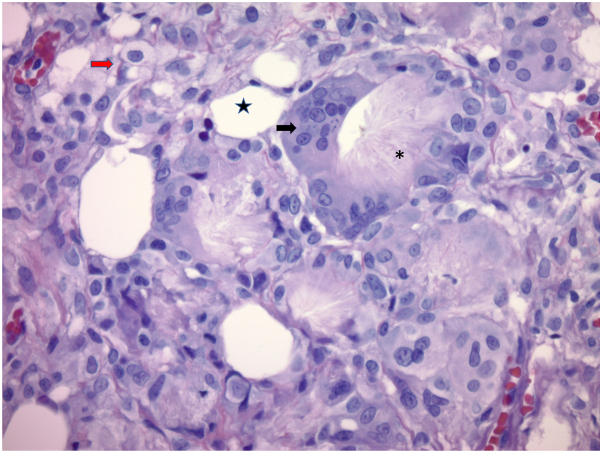
Granulomatous inflammation with foamy histiocytes (*red arrow*), multinucleated giant cells (*black arrow*), mature adipocytes (*star*), and needle shaped clefts (*asterisk*); (H&E, ×400).

A 14-day-old male infant born at 39 weeks and 1 day was seen in the dermatology clinic for evaluation of a lesion on his back. He was delivered at an outside institution and his birth history was reported to have been uncomplicated. His mother first noticed the lesion on his second day of life and felt that it had decreased in size by the time he was seen in clinic. On examination, he was found to have a 1.5 cm well circumscribed, hyperpigmented, slightly yellow, fluctuant subcutaneous nodule that blanched with the application of pressure (Fig 1). Punch biopsy was performed and resulted in the expression of milky white material, prompting concern for an infectious etiology. The histopathologic features demonstrated subcutaneous fat necrosis with radiating crystalline material within necrotic adipocytes, along with granulomatous inflammation and multinucleated giant cells, consistent with SCFN (Figs 2 and 3). Serum calcium levels monitored at 3 and 4 months of age were normal.

### Case 2

**Fig 4:**
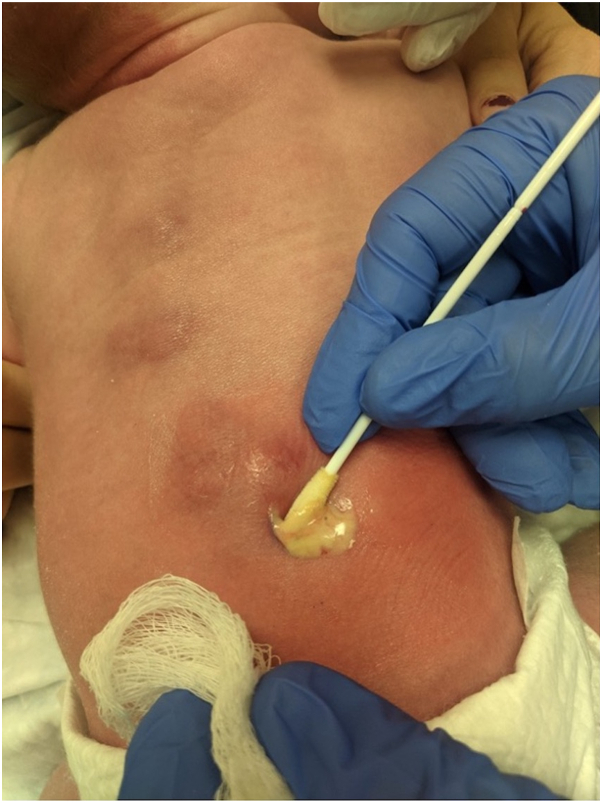
Multiple fluctuant nodules, 1 with purulent drainage following biopsy, on the back of an 11-day-old infant.

An 11-day-old otherwise healthy baby girl born full-term at 41 weeks was seen in the dermatology clinic for evaluation of 3 red nodules on her back, which appeared at day of life 3 3. The nodules were spongy and fluctuant to palpation. A 4 mm punch biopsy was attempted resulting in ‘purulent’ drainage from which a bacterial culture was obtained (Fig 4). The infant was hospitalized and received a sepsis evaluation which did not reveal a source of infection (pancultures of the blood, urine, wound drainage, and cerebrospinal fluid were negative). She remained afebrile. Three days later, the nodules on the back had decreased in size and were firm to palpation. A second 4 mm biopsy was performed demonstrating a dense lobular panniculitis containing multinucleated giant cells and needle-shaped clefts within necrotic adipocytes, consistent with SCFN. Serum calcium levels measured at day of life 15 were normal; the patient was then lost to follow-up.

## Discussion

SCFN is a benign and self-limiting panniculitis which may be associated with complications including hypercalcemia, thrombocytopenia, and hypoglycemia^6^^,^^7^; therefore, early detection is imperative. Diagnosis is clinical and may be confirmed via biopsy, which classically reveals a lobular panniculitis with a dense inflammatory infiltrate consisting primarily of histiocytes and multinucleated giant cells.^2^ Adipocytes containing needle-shaped clefts are often visible as well as scattered focal areas of calcification within the necrotic fat.^1^^,^^8^ A recent case report suggests that the diagnosis may also be confirmed via microscopic analysis of expressable lesion contents without fixation or staining, offering a rapid and minimally invasive alternative to biopsy; the identification of needle-shaped crystals distinguishes SCFN from an infectious process.^9^ Treatment consists mainly of supportive measures, as the cutaneous lesions typically resolve spontaneously within several weeks to months.^7^^,^^10^ Following initial appearance, patients should be monitored for potential complications, particularly hypercalcemia, for up to 6 months with regular clinical follow up.^7^^,^^8^ A 2019 review article examining trends in SCFN-associated hypercalcemia found that approximately one-half of infants ultimately did develop hypercalcemia, with most (77%) being diagnosed within 30 days of lesion development.^8^

The etiology of SCFN remains poorly understood, though it is commonly associated with adverse perinatal events such as hypoxia or hypothermia,^1^^,^^2^ and has been specifically associated with therapeutic hypothermia treatment for hypoxic-ischemic encephalopathy.^7^ One hypothesis encountered frequently in the literature postulates that the composition of neonatal adipose tissue predisposes to fat crystallization and necrosis in response to hypothermia due to its relatively high melting point.^1^^,^^6^^,^^8^^,^^10^ Maternal factors including gestational hypertension and pre-eclampsia have also been implicated in the development of SCFN.^8^^,^^10^

Here, we report 2 cases of SCFN in which infants had fluctuant nodules productive of milky white, purulent-appearing material rather than the classically described firm or indurated lesions.^1^^,^^2^^,^^8^

## Conflicts of interest

None declared.
